# Trophic specialization drives morphological evolution in sea snakes

**DOI:** 10.1098/rsos.172141

**Published:** 2018-03-28

**Authors:** Emma Sherratt, Arne R. Rasmussen, Kate L. Sanders

**Affiliations:** 1 School of Biological Sciences, The University of Adelaide, Adelaide, South Australia 5005, Australia; 2 The Royal Danish Academy of Fine Arts, Schools of Architecture, Design and Conservation, Copenhagen K, Denmark

**Keywords:** tempo and mode, ecomorphology, evolutionary rates, convergence, phenotypic evolution

## Abstract

Viviparous sea snakes are the most rapidly speciating reptiles known, yet the ecological factors underlying this radiation are poorly understood. Here, we reconstructed dated trees for 75% of sea snake species and quantified body shape (forebody relative to hindbody girth), maximum body length and trophic diversity to examine how dietary specialization has influenced morphological diversification in this rapid radiation. We show that sea snake body shape and size are strongly correlated with the proportion of burrowing prey in the diet. Specialist predators of burrowing eels have convergently evolved a ‘microcephalic’ morphotype with dramatically reduced forebody relative to hindbody girth and intermediate body length. By comparison, snakes that predominantly feed on burrowing gobies are generally short-bodied and small-headed, but there is no evidence of convergent evolution. The eel specialists also exhibit faster rates of size and shape evolution compared to all other sea snakes, including those that feed on gobies. Our results suggest that trophic specialization to particular burrowing prey (eels) has invoked strong selective pressures that manifest as predictable and rapid morphological changes. Further studies are needed to examine the genetic and developmental mechanisms underlying these dramatic morphological changes and assess their role in sea snake speciation.

## 1. Introduction

Rapidly speciating groups are found throughout the tree of life and have provided important insights into the mechanisms shaping biodiversity (e.g. [1]). Approaches to explaining the disparity in clade speciation rates often focus on morphological diversification: divergent selection on ecologically relevant traits, such as trophic or habitat specializations, might promote coexistence of species and potentially lead to reproductive isolation and speciation (e.g. [2–4]). Key morphological traits implicated in species radiations often show accelerated rates of evolution and highly replicate (convergent or parallel) origins in response to ecological opportunity (e.g. [5–8]). Here, we investigated the tempo and mode of body size and shape evolution in relation to trophic diversity in the most rapidly speciating reptiles known—the viviparous sea snakes (Hydrophiinae).

**Figure 1.**
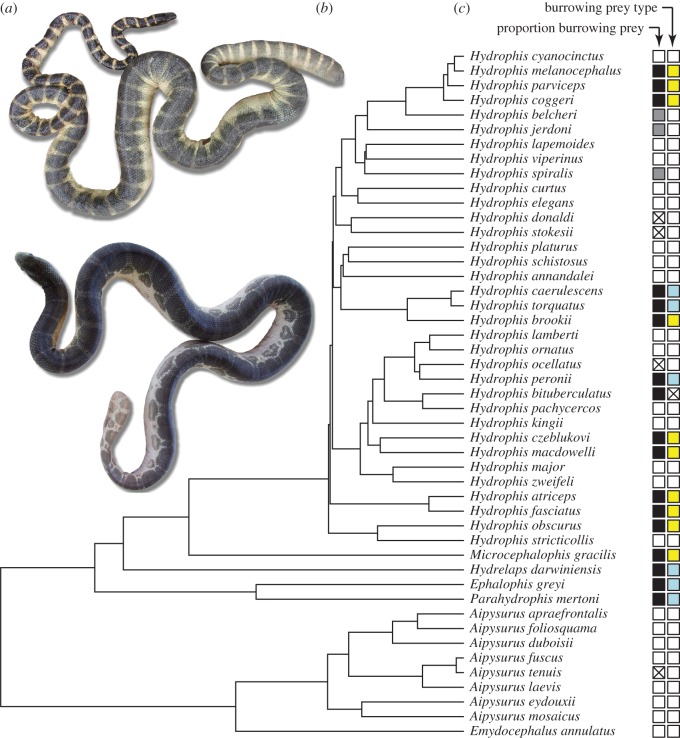
(*a*) Exemplar sea snakes showing variation in head size and forebody-hindbody proportions: top, microcephalic species, *Hydrophis atriceps* (Vietnam, photo by Arne Rasmussen), and bottom, *Hydrophis ocellatus* (Queensland, photo by Mahree-Dee White). (*b*) Maximum credibility tree of 47 species of sea snakes (see also the electronic supplementary material, figure S1). (*c*) Species were classified by the proportion of burrowing prey in their diet (white = 0–0.33, grey = 0.34–0.69, black = 0.7–1) and the primary burrowing prey type: gobies (blue) and eels (yellow). Cross indicates diet unknown.

The more than 60 species of viviparous sea snakes form eco-morphologically diverse assemblages in warm shallow-water habitats throughout the Indo-West Pacific [9,10]. Their marine origin is dated at approximately 8–17 Ma, but at least 60% of species richness in the group is accounted for by the exceptionally rapidly speciating *Hydrophis* clade—dated at 1.5–7.5 million years old [11,12]. Maximum body sizes range from 0.5 m in semi-aquatic sea snakes, to moray eel predators reaching almost 3 m. Body shape and head-body proportions are also very varied (figure 1), with the most dramatic changes found in ‘microcephalic’ sea snakes that have tiny heads and extremely narrow forebody relative to hindbody girths [9,13,14]. Sea snake body size and shape diversification have previously been linked to prey type, particularly the evolution of microcephaly in species that specialize on burrowing prey [11,14]. However, relationships between morphological and trophic diversity in sea snakes have yet to be tested in a broad-scale phylogenetic framework.

In this paper, we used a dated phylogeny for 75% of sea snake species to examine how dietary specialization has influenced morphological diversification in this rapid radiation. Specifically, we assessed: (i) the influence that proportion of burrowing prey in the diet has on sea snake body shape and size diversification, (ii) the hypothesis that dietary specialization on burrowing prey has resulted in convergent body size and shape, and (iii) whether there has been an increase in the rate of body size or shape evolution on branches leading to these burrowing prey specialists, as predicted when traits are under strong directional selection [15].

## 2. Material and methods

### 2.1. Molecular data and phylogenetic analysis

Molecular data were obtained for 47 sea snake species (GenBank numbers in Lee *et al*. [12]). Of these, 46 species were sampled in previous studies [11,12]. The newly sampled taxon was *Aipysurus tenuis*, collected in Broome, Western Australia, and identified using morphological characters following Shuntov [16]. The standard protocols used to generate mitochondrial sequences for this species are described in the electronic supplementary material.

The final alignment comprised 3972 base pairs of mitochondrial genes NADH dehydrogenase subunit 4 (ND4), cytochrome *b* (cytb) and 16S small subunit ribosomal RNA (16S rRNA), and nuclear coding genes recombination reactivating gene 1 (RAG-1) and oocyte maturation factor (c-mos). Alignment was performed using the MUSCLE plugin [17] (with translation alignment of coding sequences) in Geneious Pro v. 10.0.2 [18] and checked by eye.

Time-calibrated phylogenies were estimated using Bayesian inference in BEAST v. 2.4.7 [19]. A best-fit partitioning scheme and substitution models were determined for all possible codon positions for coding genes and all gene partitions using PartitionFinder2 [20]. The Bayesian information criterion selected five partitions of mitochondrial coding codon positions 1 + 2: GTRig; mitochondrial coding codon positions 3: GTRg; nuclear coding codon positions 1 + 2: HKYig; nuclear coding codon positions 3: HKYg; 16S rRNA: GTRig. The root node age was calibrated using a normal distribution with a mean of 16 Ma and 95% confidence intervals of 14.5 and 18.5 Ma. This is a secondary calibration that is based on the most recent molecular dating study of elapids [12].

The Markov chain Monte Carlo was run with substitution parameters unlinked across partitions for 10 000 000 generations under a Yule tree model with a uniform distribution, and an uncorrelated and lognormally distributed relaxed clock model of rate variation across adjacent branches. Tree and clock models were linked across partitions, and the chain was sampled every 10 000 generations. Convergence of the Markov chain was assessed by examining likelihood plots and histograms, and effective sample sizes (ESS values) of the estimated parameters in Tracer v. 1.6 [21]. For each run, the first 300 sampled trees were excluded as burn-in. From the remaining 700 trees, a maximum credibility tree was generated using Tree Annotator v. 2.4.6 [19] (electronic supplementary material, figure S1) and 500 trees were sampled for the statistical analyses (below).

### 2.2. Morphology

We collected data on body size and shape for 47 sea snake species sampled in our molecular analyses. For 229 individuals, average 4.8 individuals/species, we measured (to the nearest 1.0 mm using string and a ruler) girth at the neck, and girth at 0.75 snout-to-vent length (SVL), which is three-fourths down the body from the snout. Body shape was described as relative girth (girth at 0.75 SVL divided by girth at neck). Girth measurements were taken from adult males only to avoid the inclusion of gravid females carrying mature follicles or developing embryos, which would inflate the girth measurements. Individuals with obvious stomach or gut contents were also excluded. Body size was described as log-transformed maximum total length, because snakes continue to grow after reaching sexual maturity so that maximum rather than mean size provides the best estimate of age-independent adult size [22]. Total length was recorded for adults of both sexes, which were identified by large, non-flaccid testes in males and thickened oviducts and/or visible vitellogenic follicles in females. Additional body length data were collated from the literature [23–25] to estimate the maximum total length for each species. For body length data, most species were represented by very large numbers of specimens, with the exception of a few poorly known taxa. Trait data are summarized in the electronic supplementary material, tables S1 and S2.

### 2.3. Trophic ecology

New and published diet data were collated and summarized for each species (available on Dryad: http://dx.doi.org/10.5061/dryad.48r5h.2 [26]). Most records were obtained from the literature ([9,13,14] and references therein, [27–29]), and these were supplemented by snake stomach contents collected during field trips and identified to family level by relevant experts in our institutions. Diet data were used to group prey items based on prey body form (eel-like; goby-like and others, e.g. perch-like or discoid), habitat (e.g. benthic, reef) and habit (burrowing; non-burrowing—mostly crevice-sheltering), following Voris & Voris [14]. For this study, these data were used to estimate the proportion of two main types of burrowing prey (eels and gobies/goby-like fish) in each species' diet and assign these as the diet variables in the following analyses. Four species without diet records were excluded from analyses of trophic ecology with morphology: *H. ocellatus* (from Western Australia), *H. donaldi, H. stricticollis* and *A. tenuis*.

### 2.4. Statistical analyses

All statistical analyses were performed in the R statistical environment v. 3.4.2 [30] using the maximum credibility tree and (to account for phylogenetic uncertainty) 500 trees sampled from the post burn-in distribution. To examine the influence of trophic ecology on body shape and size diversification, species means for body shape and species' maximum total length were individually tested for association with diet using phylogenetic generalized least squares under a model of Brownian motion (BM) using the ‘gls’ function in *nlme* v. 3.1 [31], and the coefficient of determination (*R*^2^) estimated using a linear model of the phylogenetic independent contrasts, using the ‘pic’ function in *ape* v. 5.0 [32]. To visualize this relationship in a phylogenetic context, we used a phylomorphospace approach, whereby the consensus phylogeny was projected into a biplot of log-transformed maximum total length versus relative girth, using the independent contrasts as estimated at internal nodes.

We explicitly tested for convergence in body shape and size among the (i) 10 species of burrowing eel specialists and (ii) six species that feed predominantly on burrowing gobies and goby-like fishes. We used two approaches [33]: the first, C_5_, quantifies convergence by counting the number of convergent events, which is the number of times that a lineage has invaded a region of morphospace; the second, C_1_, measures whether the taxa have converged on a smaller area of morphospace than would be expected under BM [33]. Specifically, C_1_ relates to how much morphological divergence among extant taxa has been reduced relative to that among the estimated ancestors. Statistical significance of C_1_ was evaluated using phylogenetic simulation: the variables were simulated along the phylogeny using BM, using ‘sim.char’ function in *geiger* v. 2.0.6 [34], and the observed test measure C_1_ was compared to a distribution of 1000 simulated values using the maximum clade credibility tree. These tests were implemented using *convevol* v. 1.1 [33].

We hypothesize that a shift in diet to predominantly burrowing prey has resulted in an increased morphological rate shift along these branches. To test this hypothesis, we used maximum likelihood to fit a BM model of evolution that allows the rates of body size and body shape evolution on the phylogenetic branches leading to species of each burrowing prey group to differ from the rate in the other sea snake species. We then compared this model to a single-rate Brownian model that constrained all sea snake lineages in our sample to the same rate. This procedure follows the methods of Revell [35] and O'Meara *et al*. [36], which are implemented in the function ‘brownie.lite’ in *phytools* v. 0.6–56 [35].

## 3. Results

### 3.1. Phylogeny reconstruction

Bayesian analyses of the concatenated dataset yielded ESS values above 200 for all parameters. A total of 32 (of 48) internal nodes in the maximum credibility tree had posterior probabilities (PP) greater than 0.95. Supported relationships and branch lengths were consistent with previous concatenated mitochondrial, concatenated nuclear and multi-locus species tree analyses [11,12]. Strong support was found for all primary clades, including reciprocal monophyly of the *Aipysurus* and *Hydrophis* groups (*sensu* [23]), the placement of *Ephalophis* + *Parahydrophis* and *Hydrelaps* as successive sister lineages to the *Hydrophis* group, the relatively distant sister relationship between *Microcephalophis gracilis* and *Hydrophis*, and the *H. ornatus* and *H. cyanocinctus* groups (*sensu* [11]). Newly sampled taxon *A. tenuis* (identified using morphological characters following Shuntov [16]) was placed with strong support (PP 1.0) as a close sister lineage to *A. fuscus*. New sequences for *A. tenuis* were deposited at GenBank (accession number: MG982940).

Posterior divergence time estimates were mean 14.5 Ma and 95% highest posterior density (HPD) 11.5–17.5 Ma for the root node (all viviparous sea snakes); 7.1 Ma (95% HPD: 5.2–8.9) for the *Emydocephalus*–*Aipysurus* split; 8.6 Ma (95% HPD: 6.5–10.7) for the *Microcephalophis*–*Hydrophis* split; and 4.2 Ma (95% HPD: 3.2–5.3) for the common ancestor of all sampled *Hydrophis*. These divergence time estimates are intermediate between the deeper dates given in Lee *et al*. [12] and the shallower dates in Sanders *et al*. [11]. The timescale in Lee *et al*. [12] was calibrated directly using four fossils, whereas the Sanders *et al*. [11] analysis included only sea snakes and relied on a single secondary calibration. However, both analyses are based primarily on mitochondrial data, which could be affected by saturation along deeper internal branches in the Lee *et al*. [12] tree, resulting in moderately older age estimates for shallow nodes (e.g. within sea snakes). Future studies using a large nuclear dataset for a broad sampling of elapids will be needed to better resolve sea snake divergence times.

### 3.2. Macroevolutionary inferences

We find that trophic specialization has strongly influenced sea snake body shape and, to lesser extent, body size. While accounting for phylogenetic relationships among species, relative girth (reduced forebody relative to hindbody circumference) showed a strong positive relationship to the proportion of burrowing prey (*F*_1,41_ = 56.2, *R*^2 ^= 0.56, all 500 trees significant at *α *= 0.001), whereas total body length was negatively correlated with the proportion of burrowing prey (*F*_1,41_ = 16.5, *R*^2 ^= 0.35, all 500 trees significant at *α *= 0.05).

**Figure 2.**
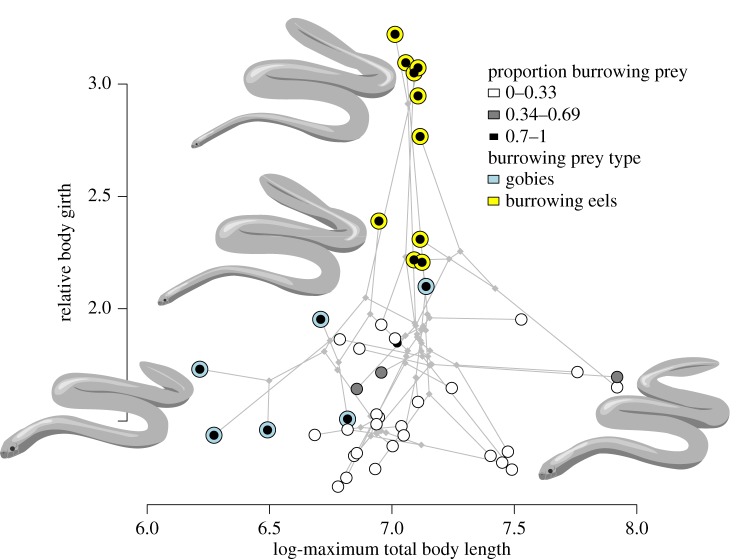
Phylomorphospace of body size versus body shape (relative girth; girth at 0.75 SVL/girth at neck) among 47 species of sea snakes. Points represent species, coloured as in figure 1. The maximum credibility tree (figure 1*b* and electronic supplementary material, S1) is projected into this morphospace using maximum-likelihood ancestral state reconstruction.

The size-shape morphospace shown in figure 2 illustrates the relationships among morphological and trophic traits. Seventeen of the 47 sampled species have diets with high proportions (greater than 0.7) of burrowing prey; six of these species feed predominantly on gobies and 10 predominantly on eels, with the two groups occupying distinctly different regions of the morphospace (figure 2). Comparing the two burrowing prey type groups and the other species, we find that body shape is substantially different in eel specialists compared to goby feeders and other species (*F*_1,39_ = 40.7, all 500 trees significant at *α *= 0.001), while body length differs between all three groups (*F*_1,39_ = 12.1, all 449/500 trees significant at *α *= 0.001; electronic supplementary material, figure S2).

There is a statistical correlation between relative girth and maximum total body length across species (*F*_1,45_ = 8.2, *R*^2^ = 0.13, all 497/500 trees significant at *α *= 0.05, slope = −0.52); however, it is clear from the morphospace that the relationship deviates from linear (figure 2). This is due specifically to the 10 species with specialist diets comprised 70–100% burrowing eels; these are all clearly separated from other sampled species along the *y*-axis, having forebody girths of at least half to more than one-third their hindbody girth (i.e. are ‘microcephalic’ *sensu* [9]), and are intermediate in maximum total body length among the sampled species in this study. The region of morphospace occupied by the eel specialists is elongate and appears to comprise two distinct clusters along the body shape axis (figure 2); six species are strongly microcephalic with relative girths of 2.7–3.2 (*H. atriceps, H. fasciatus, H. parviceps, H. macdowelli, H. obscurus, M. gracilis*), and four species are moderately microcephalic with relative girths of 2.2–2.4 (*H. coggeri, H. melanocephalus, H. brooki, H. czeblukovi*). None of the sampled species have a relative girth of 2.5–2.7.

We find evidence of strong convergent evolution among the 10 species of eel specialists: first, using a frequency-based measure of convergence, the number of times that a lineage has invaded this ‘microcephalic’ region of morphospace is nine (convergence measure C_5_, derived from the maximum credibility tree; mean from 500 trees = 8.7 ± 0.49). Second, the similarity-based measure of convergence C_1_ is 0.55, implying that evolution has closed 55% of the distance between the eel specialists in the size-shape morphospace. This was a much smaller area of morphospace than expected under a BM model of evolution (*p* < 0.001). By comparison, we do not find evidence of convergent evolution among the six species that feed on gobies (*C*_5_ = 1; *C*_1_* *= 0.23, *p *= 0.103).

Dietary specialization of burrowing prey has also coincided with a marked shift in the rates of morphological diversification for eel specialists but not for species that feed predominantly on gobies: both relative girth and maximum total body length have evolved significantly faster in eel specialists compared with any other species (relative girth: *σ*^2^_eel_ = 0.56, *σ*^2^_goby_ = 0.14, *σ*^2^_other_ = 0.015, all 500 trees significant at *α *= 0.05; body length: *σ*^2^_eel_ = 0.15, *σ*^2^_goby_ = 0.012, *σ*^2^_other_ = 0.036, all 500 trees significant at *α *= 0.05; electronic supplementary material, figure S3).

## 4. Discussion

Our study showed that sea snake body shape and size are highly variable and strongly correlated with trophic ecology, specifically the proportion of burrowing prey. Most notable in the size-shape morphospace are 10 species that specialize on burrowing eel prey. These ‘microcephalic’ species have dramatically reduced forebody relative to hindbody girths and are morphologically evolving in a unique manner compared with other sea snakes (figure 2). Firstly, they appear to have broken away and subsequently diversified from other species along the body shape axis. The microcephalic species form an elongate cluster, yet six or seven of the 10 lineages are derived from non-microcephalic ancestors, revealing important roles for convergent evolution and directional selection in response to trophic opportunity. Also notable within this cluster are distinct groups of six strongly microcephalic versus four moderately microcephalic species. This observation does not appear to be explained by lineage age; strongly microcephalic species do not typically show deeper divergence times from non-microcephalic ancestors compared with moderately microcephalic species (electronic supplementary material, figure S1). Hence, the two groups may represent two distinct optima in the adaptive landscape (e.g. [37]). Future studies should aim to assess this hypothesis by examining the three microcephalic species missing from our dataset (*Hydrophis klossi*, *H. mamillaris* and *Microcephalophis cantoris*), and better characterizing trophic diversity within the group (e.g. by distinguishing types of burrowing eel prey).

There is a compelling functional explanation for microcephaly in burrowing prey specialists: narrow heads and forebodies allow these snakes to probe fish burrows on the sea floor, a behaviour that has been observed in sea snakes that prey on eels [27,38,39] and gobies [40]. However, in contrast to the burrowing eel specialists, species that feed predominantly on burrowing gobies do not converge in the size-shape morphospace: at least four of the six species are non-microcephalic and among the shortest snakes sampled, yet the girth dimensions of their forebodies are similar to those of the microcephalic species (electronic supplementary material, table S2). Having a relatively standard snake shape (relative girth less than 2) but reducing overall body size may be an alternative solution for having heads and forebodies narrow enough to probe burrows. However, given that microcephly involves a decoupling of head/forebody and hindbody development, the observation that this is a more common evolutionary event warrants explanation. Longer bodies with thick hindbody girths probably allow larger prey to be taken, but it is also reasonable to expect that larger snakes can dive to deeper habitats in search of prey. Consistent with the latter prediction is that three of the four non-microcephalic goby predators occupy very shallow inshore habitats and forage in burrows on exposed banks [41,42].

More generally, costs to locomotor performance may constrain body shape evolution more tightly in terrestrial and amphibious snakes compared with fully aquatic snakes [43]. Species that hunt in burrows are found throughout snake phylogeny and some are highly specialist in this behaviour [44–46]. However, it is notable that no other lineage of fully aquatic snakes has evolved microcephaly given similar ecological opportunity provided by the near ubiquitous presence of burrowing fish prey in shallow marine habitats. In particular, *Aipysurus* and *Emydocephalus* share many habitats with *Hydrophis* and have diversified over a similar time frame [10], yet none of these species is microcephalic or known to heavily exploit burrowing eel prey. This suggests an intrinsic propensity of *Hydrophis* to rapidly respond to the availability of prey resources by evolving head size and body shape changes. An important remaining question is whether the remarkably frequent origins of microcephaly in *Hydrophis* are owing to recurrent (de novo) mutations in multiple lineages, or alternatively stem from standing (pre-existing) genetic variation [47]. Future studies are needed to distinguish these hypotheses, but the convergent evolution from standing variation may be more likely given the very narrow intervals between successive speciation events in *Hydrophis* [11].

Finally, rapid body shape evolution in response to prey availability is a plausible driver of the anomalously high rates of diversification in *Hydrophis.* Microcephalic lineages account for approximately 30% of species richness in *Hydrophis*, and both microcephalic and non-microcephalic populations are found within at least five other *Hydrophis* species (not represented in our macroevolutionary analysis). By sampling 175 species of elapids, Lee *et al*. [12] showed that *Hydrophis* have highly elevated rates of species diversification compared with other (primarily terrestrial) snakes, but this was not correlated with a corresponding rate shift in body size evolution. Unfortunately, there are too few species of sea snakes with which to also test for a relationship between species diversification rates and rates of body shape evolution (e.g. [4,48]). However, our results highlight the importance of using a diverse array of morphological data, beyond simply size, to understand ecological drivers of species diversification.

## 5. Conclusion

Our study has revealed that trophic specialization has had a strong influence on body morphology in sea snakes, and this relationship is predominantly driven by the convergent evolution of microcephalic burrowing eel specialists. Dietary specialization appears to invoke strong selective pressures that manifest as predictable and rapid morphological changes. Future studies are needed to examine the genetic and developmental mechanisms underlying these dramatic body shape changes and address their role in speciation.

## Supplementary Material

Supplementary Materials

## Data accessibility

Morphological and diet data, phylogenetic trees and R scripts are available on Dryad, http://dx.doi.org/10.5061/dryad.48r5h.2 [26]).
