# Supplementary material for: Trophic specialization drives morphological evolution in sea snakes
Source: R Soc Open Sci. 2018 Mar 28;5(3):172141. doi: 10.1098/rsos.172141 (PMC5882731; doi:10.1098/rsos.172141)

**Supplementary Materials and Methods**

**Molecular methods.**

Genomic DNA was extracted from ethanol-preserved tissues using a Puregene™ DNA Isolation Tissue Kit (Gentra Systems). 1104 base pairs of the mitochondrial cytochrome b gene was amplified by PCR in 25 μL volumes using Immolase Taq polymerase (Bioline) and primers from Burbrink et al. 2000. Double-stranded sequencing was outsourced to the Australian Genome Research Facility Ltd. (AGRF) in Adelaide, Australia. Sequences were checked for ambiguities, and alignments were assembled from consensus sequences of forward and reverse reads using Geneious Pro v.10.0.2 (Kearse et al. 2012).

**References**

Burbrink, F.T., Lawson, R., Slowinski, J.P., 2000. Mitochondrial DNA phylogeography of the polytypic North American rat snake (Elaphe obsoleta): a critique of the subspecies concept. Evolution 54, 2107–2118.

Kearse M., Moir R., Wilson A., Stones-Havas S., Cheung M., Sturrock S., Buxton S., Cooper A., Markowitz S., Duran C., et al. 2012. Geneious Basic: an integrated and extendable desktop software platform for the organization and analysis of sequence data. Bioinformatics 28(12), 1647-1649. (doi:10.1093/bioinformatics/bts199).

**Table S1** Maximum total body length for 47 sea snake species. References given below the table

| Species | Max total length (mm) | Reference |
| --- | --- | --- |
| *Aipysurus apraefrontalis* | 1040 | Rasmussen and Sanders, pers. obs. |
| *Aipysurus duboisii* | 1140 | Rasmussen and Sanders, pers. obs. |
| *Aipysurus eydouxii* | 915 | Sanders et al 2012 |
| *Aipysurus foliosquama* | 800 | Rasmussen and Sanders, pers. obs. |
| *Aipysurus fuscus* | 940 | Rasmussen and Sanders, pers. obs. |
| *Aipysurus laevis* | 1720 | Rasmussen and Sanders, pers. obs. |
| *Aipysurus mosaicus* | 1100 | Rasmussen and Sanders, pers. obs. |
| *Aipysurus tenuis* | 1030 | Rasmussen and Sanders, pers. obs. |
| *Emydocephalus annulatus* | 1030 | Rasmussen and Sanders, pers. obs. |
| *Ephalophis greyi* | 660 | Rasmussen and Sanders, pers. obs. |
| *Hydrelaps darwiniensis* | 530 | Rasmussen and Sanders, pers. obs. |
| *Hydrophis annandalei* | 910 | Rasmussen and Sanders, pers. obs. |
| *Hydrophis atriceps* | 1220 | Rasmussen and Sanders, pers. obs. |
| *Hydrophis belcheri* | 950 | Rasmussen and Sanders, pers. obs. |
| *Hydrophis bituberculatus* | 1120 | Rasmussen and Sanders, pers. obs. |
| *Hydrophis brookii* | 1040 | Rasmussen and Sanders, pers. obs. |
| *Hydrophis caerulescens* | 980 | Rasmussen and Sanders, pers. obs. |
| *Hydrophis coggeri* | 1200 | Rasmussen and Sanders, pers. obs. |
| *Hydrophis curtus* | 1100 | Rasmussen and Sanders, pers. obs. |
| *Hydrophis cyanocinctus* | 2750 | Rasmussen and Sanders, pers. obs. |
| *Hydrophis czeblukovi* | 1240 | Rasmussen and Sanders, pers. obs. |
| *Hydrophis donaldi* | 886 | Ukuwela et al 2012 |
| *Hydrophis elegans* | 2340 | Rasmussen and Sanders, pers. obs. |
| *Hydrophis fasciatus* | 1110 | Das 2010 |
| *Hydrophis jerdoni* | 1050 | Rasmussen and Sanders, pers. obs. |
| *Hydrophis kingii* | 1860 | Rasmussen and Sanders, pers. obs. |
| *Hydrophis lamberti* | 1220 | Rasmussen and Sanders, pers. obs. |
| *Hydrophis lapemoides* | 960 | Das 2010 |
| *Hydrophis macdowelli* | 1160 | Rasmussen and Sanders, pers. obs. |
| *Hydrophis major* | 1640 | Rasmussen and Sanders, pers. obs. |
| *Hydrophis melanocephalus* | 1230 | Rasmussen and Sanders, pers. obs. |
| *Hydrophis obscurus* | 1200 | Rasmussen and Sanders, pers. obs. |
| *Hydrophis ocellatus* | 1365 | Rasmussen and Sanders, pers. obs. |
| *Hydrophis ornatus* | 1150 | Das 2010 |
| *Hydrophis pachycercos* | 1110 | Rasmussen et al 2007 |
| *Hydrophis parviceps* | 1230 | Rasmussen et al 2012 |
| *Hydrophis peronii* | 1260 | Rasmussen and Sanders, pers. obs. |
| *Hydrophis platurus* | 880 | Rasmussen and Sanders, pers. obs. |
| *Hydrophis schistosus* | 1400 | Rasmussen and Sanders, pers. obs. |
| *Hydrophis spiralis* | 2750 | Das 2010 |
| *Hydrophis stokesii* | 1790 | Rasmussen and Sanders, pers. obs. |
| *Hydrophis stricticollis* | 1050 | Das 2010 |
| *Hydrophis torquatus* | 915 | Rasmussen and Sanders, pers. obs. |
| *Hydrophis viperinus* | 950 | Rasmussen and Sanders, pers. obs. |
| *Hydrophis zweifeli* | 1024 | Rasmussen and Sanders, pers. obs. |
| *Microcephalophis gracilis* | 1220 | Rasmussen and Sanders, pers. obs. |
| *Parahydrophis mertoni* | 500 | Rasmussen and Sanders, pers. obs. |

**References**

Das, I. 2010. Reptiles of South-East Asia. New Holland Publishers (UK), London, 376 pp.

Rasmussen, A.R., Gravlund, P., Van Nguyen, C. and Chanhome, L. 2007. A resurrection of Hydrophis pachycercos Fischer 1855 (Serpentes: Elapidae) with a new neotype from the South China Sea. Hamadryad 31(2): 288-298.

Rasmussen A.R., Elmberg J., Sanders K.L., Gravlund P. 2012 Rediscovery of the Rare Sea Snake Hydrophis parviceps Smith 1935: Identification and Conservation Status. Copeia: June 2012, Vol. 2012, No. 2, pp. 276-282.

Sanders KL, Rasmussen AR, Elmberg J, Mumpuni, Guinea M, Blias P, Lee MSY, Fry BG (2012) Aipysurus mosaicus, a new species of egg-eating sea snake (Elapidae: Hydrophiinae), with a redescription of Aipysurus eydouxii (Gray, 1849). Zootaxa 3431: 1-18.

Ukuwela, K D. B., Sanders, KL., Fry, BG. (2012): *Hydrophis donaldi* (Elapidae: Hydrophiinae), a highly distinctive new species of sea snake from northern Australia. Zootaxa 3201: 45-57.

**Table S2** Body shape data for 47 sea snake species (228 specimens). Relative girth was calculated as the ratio between the girth at 0.75 snout-to-vent length (SVL) and the girth at neck. Specimen IDs refer to museum collections from the following museums: Australian Museum Sydney (AMS), Field Museum of Natural History (FMNH), Museum Zoologicum Bogoriense (MZB), Natural History Museum, London (BMNH), Queensland Museum (QM), South Australia Museum (SAM), Western Australia Museum (WAM), and Zoological Museum -University of Copenhagen (ZMUC). KLS is a field label for unaccessioned specimens.

| Species | Locality | Specimen ID number | Girth at Neck | Girth at 0.75 SVL |
| --- | --- | --- | --- | --- |
| *Acalyptophis peronii* | Vietnam | ZMUC661208 | 7 | 13.5 |
| *Acalyptophis peronii* | Vietnam | ZMUC661330 | 5 | 9.6 |
| *Acalyptophis peronii* | Vietnam | ZMUC661331 | 6.3 | 12 |
| *Acalyptophis peronii* | Vietnam | ZMUC661431 | 4.9 | 9.8 |
| *Acalyptophis peronii* | Australia | FMNH213670 | 4.1 | 8.1 |
| *Acalyptophis peronii* | Australia | WAMR83950 | 3.5 | 9.4 |
| *Acalytophis peronii* | Australia | WAMR73656 | 4.3 | 9.2 |
| *Acalytophis peronii* | Australia | WAMR59787 | 4.6 | 10.3 |
| *Aipysurus aparefrontalis* | Australia | QdM80569 | 6 | 8 |
| *Aipysurus apraefrontalis* | Australia | WAMR26716 | 4.1 | 6.4 |
| *Aipysurus apraefrontalis* | Australia | BMNH 1946.1.1.95 | 4.2 | 5.6 |
| *Aipysurus apraefrontalis* | Australia | BMNH 1946.1.1.94 | 3.7 | 6.8 |
| *Aipysurus duboisii* | Australia | ZMUC661229 | 6.2 | 10.2 |
| *Aipysurus duboisii* | Australia | QdM80494 | 7.2 | 10.5 |
| *Aipysurus duboisii* | Australia | QdM80495 | 5.3 | 6.9 |
| *Aipysurus duboisii* | Australia | WAMR156216 | 5.5 | 8.7 |
| *Aipysurus duboisii* | Australia | WAMR154751 | 5 | 7 |
| *Aipysurus eydouxii* | Singapore | SAMR22569 | 3.9 | 5.7 |
| *Aipysurus foliosquama* | Australia | FMNH216509 | 4.9 | 6.8 |
| *Aipysurus foliosquama* | Australia | WAMR129806 | 4.5 | 6.7 |
| *Aipysurus fuscus* | Australia | WAMR129816 | 5.5 | 7 |
| *Aipysurus fuscus* | Australia | WAMR129815 | 6 | 9.4 |
| *Aipysurus fuscus* | Australia | BMNH 1926.11.22 | 4.9 | 6.3 |
| *Aipysurus fuscus* | Australia | BMNH 1926.11.21 | 4.3 | 5.4 |
| *Aipysurus laevis* | Australia | WAMR23635 | 8.7 | 10.8 |
| *Aipysurus laevis* | Australia | WAMR29931 | 6.4 | 8.1 |
| *Aipysurus laevis* | Australia | WAMR73659 | 6.4 | 10.2 |
| *Aipysurus laevis* | Australia | WAMR73672 | 10.1 | 12 |
| *Aipysurus laevis* | Australia | WAMR98799 | 8 | 10.3 |
| *Aipysurus mosaicus* | Australia | SAMR22569 | 3.7 | 5.5 |
| *Aipysurus mosaicus* | Australia | SAMR65222 | 3.9 | 5.5 |
| *Aipysurus mosaicus* | Coral Sea | BMNH 1928.4.11.5 | 3.1 | 3.8 |
| *Aipysurus mosaicus* | Australia | QMJ67313 | 5.6 | 8 |
| *Aipysurus tenuis* | Broome, WA | ZMUC661451 | 5.5 | 8.5 |
| *Aipysurus tenuis* | Broome, WA | KLS0654 | 5 | 7.5 |
| *Aipysurus tenuis* | Broome, WA | KLS0657 | 4.8 | 7.4 |
| *Emydocephalus annulatus* | Australia | QdM80685 | 4.5 | 6.1 |
| *Emydocephalus annulatus* | Australia | ZMUC661198 | 4.2 | 6 |
| *Emydocephalus annulatus* | Australia | WAMR47852 | 4.3 | 7 |
| *Emydocephalus annulatus* | Australia | WAMR73651 | 6.2 | 9.5 |
| *Ephalophis greyi* | Australia | WAMR112180 | 2.5 | 3.1 |
| *Ephalophis greyi* | Australia | WAMR157940 | 2.2 | 3.2 |
| *Ephalophis greyi* | Australia | WAMR112179 | 2.3 | 3.1 |
| *Ephalophis greyi* | Australia | QMJ51993 | 3.5 | 6.2 |
| *Ephalophis greyi* | Australia | SAMR35077 | 3.3 | 4.9 |
| *Hydrelaps darwinensis* | Australia | SAMR2270.D | 2.4 | 3.3 |
| *Hydrelaps darwinensis* | Australia | SAMR2270.C | 2.3 | 3.3 |
| *Hydrelaps darwinensis* | Australia | WAMR22346 | 3 | 4.5 |
| *Hydrophis czeblukovi* | Broome, WA | ZMUC661437 | 5.8 | 14 |
| *Hydrophis kingii* | Australia | QMJ80500 | 4.9 | 10.5 |
| *Hydrophis kingii* | Australia | QMJ79323 | 5.2 | 9.7 |
| *Hydrophis annandalei* | Vietnam | ZMUC | 8 | 10.5 |
| *Hydrophis annandalei* | Thailand | FMNH179041 | 4 | 4.9 |
| *Hydrophis annandalei* | Vietnam | BMNH1921.2.11.7 | 4.9 | 5.7 |
| *Hydrophis annandalei* | Thailand | BMNH 1903.4.13.80 | 4.5 | 5.8 |
| *Hydrophis atriceps* | Malaysia | FMNH198826 | 1.3 | 5.4 |
| *Hydrophis atriceps* | Malaysia | FMNH198831 | 2 | 5.3 |
| *Hydrophis atriceps* | Malaysia | FMNH199263 | 2 | 5.7 |
| *Hydrophis atriceps* | Malaysia | FMNH199320 | 2 | 5.3 |
| *Hydrophis atriceps* | Malaysia | FMNH199329 | 2.2 | 5.5 |
| *Hydrophis atriceps* | Malaysia | FMNH201969 | 1.9 | 5.8 |
| *Hydrophis atriceps* | Malaysia | FMNH202049 | 2.2 | 6.1 |
| *Hydrophis belcheri* | Vietnam | ZMUC661289 | 5.7 | 10.5 |
| *Hydrophis belcheri* | Vietnam | ZMUC661212 | 4.5 | 6.5 |
| *Hydrophis belcheri* | Vietnam | ARR3426 | 3.5 | 6.8 |
| *Hydrophis belcheri* | Java Sea | BMNH 1977.125 | 4.1 | 5.5 |
| *Hydrophis bituberculatus* | Phuket Port | ZMUC66688 | 5.4 | 10.6 |
| *Hydrophis bituberculatus* | Phuket Port | ZMUC66690 | 5.6 | 10.8 |
| *Hydrophis bituberculatus* | Phuket Port | ZMUC66691 | 5 | 8.9 |
| *Hydrophis bituberculatus* | Phuket Port | ZMUC66692 | 5.5 | 10.3 |
| *Hydrophis bituberculatus* | Phuket Port | ZMUC66695 | 5.3 | 10.3 |
| *Hydrophis bituberculatus* | Phuket Port | ZMUC66696 | 4.8 | 8.6 |
| *Hydrophis bituberculatus* | Phuket Port | ZMUC66697 | 5.9 | 9.7 |
| *Hydrophis bituberculatus* | Phuket Port | ZMUC66769 | 5.1 | 9.9 |
| *Hydrophis bituberculatus* | Phuket Port | ZMUC66770 | 5.5 | 10.4 |
| *Hydrophis bituberculatus* | Phuket Port | ZMUC66771 | 5.4 | 9.3 |
| *Hydrophis brookii* | Malaysia | FMNH198613 | 2.5 | 5.2 |
| *Hydrophis brookii* | Malaysia | FMNH199593 | 1.9 | 4.9 |
| *Hydrophis brookii* | Malaysia | FMNH199594 | 2.1 | 5.5 |
| *Hydrophis brookii* | Malaysia | FMNH199597 | 2.2 | 4.8 |
| *Hydrophis brookii* | Malaysia | FMNH201180 | 2.1 | 5.6 |
| *Hydrophis brookii* | Malaysia | FMNH201364 | 2.4 | 5.6 |
| *Hydrophis brookii* | Malaysia | FMNH203403 | 2.9 | 6.7 |
| *Hydrophis caerulescens* | Malaysia | FMNH198938 | 2.7 | 4.8 |
| *Hydrophis caerulescens* | Malaysia | FMNH198955 | 3.2 | 6.3 |
| *Hydrophis caerulescens* | Malaysia | FMNH199250 | 2.7 | 5.5 |
| *Hydrophis caerulescens* | Malaysia | FMNH199253 | 3.4 | 6.1 |
| *Hydrophis caerulescens* | Malaysia | FMNH199258 | 2.7 | 5.5 |
| *Hydrophis caerulescens* | Malaysia | FMNH199260 | 2.9 | 5.9 |
| *Hydrophis caerulescens* | Malaysia | FMNH201153 | 3.1 | 5.5 |
| *Hydrophis caerulescens* | Malaysia | FMNH202054 | 3 | 6.6 |
| *Hydrophis coggeri* | Sulawesi | KLS_MT166 | 26 | 60 |
| *Hydrophis coggeri* | Sulawesi | KLS_MT162 | 35 | 75 |
| *Hydrophis coggeri* | Sulawesi | KLS_MT174 | 50 | 110 |
| *Hydrophis curtus* | Malaysia | FMNH201910 | 7 | 9.9 |
| *Hydrophis curtus* | Malaysia | FMNH202011 | 7.7 | 10.1 |
| *Hydrophis curtus* | Malaysia | FMNH202021 | 5.7 | 7.1 |
| *Hydrophis curtus* | Malaysia | FMNH202030 | 6.8 | 10.3 |
| *Hydrophis curtus* | Malaysia | FMNH202032 | 6.3 | 8.4 |
| *Hydrophis cyanocinctus* | Java | KLS_SM063 | 5.7 | 8.5 |
| *Hydrophis cyanocinctus* | Java | KLS_MW04634 | 5.5 | 9 |
| *Hydrophis cyanocinctus* | Java | KLS_MW04636 | 5 | 8.5 |
| *Hydrophis cyanocinctus* | Java | MZB3893 | 4.7 | 8.5 |
| *Hydrophis cyanocinctus* | Java | KLS_MW04632 | 6.5 | 10.5 |
| *Hydrophis czeblukovi* | Australia | WAMR102516 | 4 | 8 |
| *Hydrophis donaldi* | Australia | SAMR66274 | 3 | 6 |
| *Hydrophis donaldi* | Australia | SAMR65216 | 3.3 | 5.7 |
| *Hydrophis elegans* | Gulf of Carpentaria | QMJ84492 | 5.5 | 8.7 |
| *Hydrophis elegans* | Gulf of Carpentaria | QMJ83621 | 6.1 | 11.2 |
| *Hydrophis elegans* | Gulf of Carpentaria | QMJ83704 | 5.1 | 9.8 |
| *Hydrophis elegans* | Gulf of Carpentaria | QMJ81616 | 6.5 | 9.7 |
| *Hydrophis elegans* | Gulf of Carpentaria | QMJ81707 | 4.2 | 7.5 |
| *Hydrophis elegans* | Gulf of Carpentaria | QMJ82248 | 6.6 | 10.7 |
| *Hydrophis elegans* | Gulf of Carpentaria | QMJ79493 | 5.3 | 10.5 |
| *Hydrophis elegans* | Gulf of Carpentaria | QMJ80541 | 4.6 | 7 |
| *Hydrophis elegans* | Gulf of Carpentaria | QMJ80840 | 5.2 | 8.3 |
| *Hydrophis elegans* | Gulf of Carpentaria | QMJ80888 | 5.2 | 9.6 |
| *Hydrophis fasciatus* | *?* | SAMR147143 | 1.8 | 5.8 |
| *Hydrophis jerdoni* | Phuket Thailand | ZMUC66700 | 4.5 | 7.4 |
| *Hydrophis jerdoni* | Phuket Thailand | ZMUC66701 | 4.2 | 7 |
| *Hydrophis jerdoni* | Phuket Thailand | ZMUC66706 | 4.8 | 8.7 |
| *Hydrophis jerdoni* | Phuket Thailand | ZMUC66707 | 4.5 | 7.8 |
| *Hydrophis jerdoni* | Phuket Thailand | ZMUC66763 | 4.5 | 7.5 |
| *Hydrophis jerdoni* | Phuket Thailand | ZMUC66766 | 4.2 | 7 |
| *Hydrophis jerdoni* | Thailand | FMNH178769 | 3.8 | 5.8 |
| *Hydrophis jerdoni* | Thailand | FMNH242107 | 4.5 | 8.4 |
| *Hydrophis jerdoni* | Thailand | FMNH242143 | 3.6 | 6.7 |
| *Hydrophis kingii* | Australia | WAMR71739 | 4.6 | 8.5 |
| *Hydrophis lamberti* | Gulf of Thailand | ZMUC661129 | 7.5 | 13.1 |
| *Hydrophis lamberti* | Gulf of Thailand | ZMUC661200 | 7.1 | 10.1 |
| *Hydrophis lapemoides* | Phuket, Thailand | ZMUC66881 | 4.5 | 7.8 |
| *Hydrophis lapemoides* | Phuket, Thailand | ZMUC66888 | 4.5 | 7.6 |
| *Hydrophis lapemoides* | Phuket, Thailand | ZMUC66889 | 3.6 | 7.4 |
| *Hydrophis lapemoides* | Phuket, Thailand | ZMUC66588 | 3.8 | 6.5 |
| *Hydrophis lapemoides* | Phuket, Thailand | ZMUC66589 | 4.5 | 7.5 |
| *Hydrophis lapemoides* | Phuket, Thailand | ZMUC66592 | 3.5 | 7.8 |
| *Hydrophis lapemoides* | Phuket, Thailand | ZMUC66597 | 4 | 8 |
| *Hydrophis lapemoides* | Phuket, Thailand | ZMUC66598 | 4 | 7.2 |
| *Hydrophis lapemoides* | Phuket, Thailand | ZMUC66600 | 4.7 | 6.8 |
| *Hydrophis lapemoides* | Phuket, Thailand | ZMUC66891 | 4.5 | 7.5 |
| *Hydrophis lapemoides* | Phuket, Thailand | ZMUC66895 | 4.2 | 7.8 |
| *Hydrophis lapemoides* | Phuket, Thailand | ZMUC66894 | 3.5 | 5.9 |
| *Hydrophis lapemoides* | Phuket, Thailand | ZMUC66894 | 3.5 | 5.9 |
| *Hydrophis lapemoides* | Phuket, Thailand | ZMUC66896 | 3.7 | 7 |
| *Hydrophis lapemoides* | Phuket, Thailand | ZMUC661091 | 3.4 | 7.5 |
| *Hydrophis macdowelli* | Broome, Australia | ZMUC661430 | 3.1 | 11.5 |
| *Hydrophis macdowelli* | Broome, Australia | ZMUC661429 | 3.1 | 10.1 |
| *Hydrophis macdowelli* | Australia | QMJ80570 | 3 | 7.5 |
| *Hydrophis macdowelli* | Australia | AMR14425 | 2.5 | 7.6 |
| *Hydrophis macdowelli* | Australia | QMJ42473 | 2.9 | 8.6 |
| *Hydrophis major* | Australia | SAMR29380 | 6.3 | 8 |
| *Hydrophis major* | Australia | WAMR44392 | 6.2 | 7.9 |
| *Hydrophis major* | Australia | AMR110312 | 5.5 | 7.7 |
| *Hydrophis major* | Australia | WAMR174253 | 7.4 | 10.5 |
| *Hydrophis major* | Australia | WAMR174252 | 7.5 | 10.2 |
| *Hydrophis melanocephalus* | Vietnam | ZMUC-X01036 | 3.3 | 7 |
| *Hydrophis melanocephalus* | Vietnam | ZMUC-X01037 | 3.3 | 6.8 |
| *Hydrophis melanocephalus* | Vietnam | ZMUC-X01038 | 3 | 7.5 |
| *Hydrophis melanocephalus* | Vietnam | ZMUC-X01039 | 3.2 | 7.3 |
| *Hydrophis melanocephalus* | Vietnam | ZMUC-X01041 | 3.5 | 8.3 |
| *Hydrophis melanocephalus* | Vietnam | ZMUC-X01044 | 3.7 | 8.4 |
| *Hydrophis melanocephalus* | Vietnam | ZMUC-X01045 | 3.4 | 8.8 |
| *Hydrophis obscurus* | Bangladesh | ZMUC661302 | 2.4 | 6.1 |
| *Hydrophis obscurus* | Bangladesh | ZMUC661304 | 2.1 | 7 |
| *Hydrophis obscurus* | Bangladesh | ZMUC661310 | 2 | 6.9 |
| *Hydrophis obscurus* | Bangladesh | ZMUC661317 | 2.2 | 6.8 |
| *Hydrophis obscurus* | Bangladesh | ZMUC661319 | 2 | 5.4 |
| *Hydrophis obscurus* | Bangladesh | ZMUC661323 | 1.6 | 5.2 |
| *Hydrophis obscurus* | Bangladesh | ZMUC661324 | 1.9 | 5 |
| *Hydrophis obscurus* | Bangladesh | ZMUC661327 | 2.2 | 7 |
| *Hydrophis obscurus* | Bangladesh | ZMUC661328 | 2.2 | 7.2 |
| *Hydrophis ocellatus* | Western Australia | WAMR174535 | 5.8 | 11.4 |
| *Hydrophis ocellatus* | Western Australia | WAMR174523 | 5 | 11.5 |
| *Hydrophis ocellatus* | Western Australia | WAMR174533 | 5.7 | 11.5 |
| *Hydrophis ornatus* | Philippines | FMNH202860 | 4.4 | 6.3 |
| *Hydrophis ornatus* | Philippines | FMNH202863 | 5.6 | 8 |
| *Hydrophis ornatus* | Philippines | FMNH202865 | 5.7 | 9.1 |
| *Hydrophis ornatus* | Malaysia | FMNH257228 | 7.8 | 9.4 |
| *Hydrophis ornatus* | Malaysia | FMNH257229 | 6.1 | 8.4 |
| *Hydrophis ornatus* | Malaysia | FMNH257233 | 6.2 | 9.2 |
| *Hydrophis ornatus* | Malaysia | FMNH257236 | 5.5 | 8.4 |
| *Hydrophis pachycercos* | Vietnam | ZMUCR661232/X01009 | 5.3 | 9.2 |
| *Hydrophis pachycercos* | Vietnam | ZMUCR661231/X01047 | 5.6 | 11.2 |
| *Hydrophis parviceps* | Vietnam | ZMUCR661281 | 3 | 8.5 |
| *Hydrophis platurus* | Ecuador | FMNH16927 | 5.5 | 6.8 |
| *Hydrophis platurus* | Panama | FMNH37376 | 5.5 | 6.7 |
| *Hydrophis platurus* | Peru | FMNH41591 | 5.4 | 7 |
| *Hydrophis platurus* | Costa Rica | FMNH171606 | 4.1 | 4.8 |
| *Hydrophis platurus* | Costa Rica | FMNH171610 | 4.6 | 5.6 |
| *Hydrophis platurus* | Costa Rica | FMNH171674 | 4.5 | 5 |
| *Hydrophis schistosus* | Malaysia | FMNH198478 | 4.2 | 7.8 |
| *Hydrophis schistosus* | Malaysia | FMNH198486 | 5.2 | 10 |
| *Hydrophis schistosus* | Malaysia | FMNH198487 | 5 | 8.6 |
| *Hydrophis schistosus* | Malaysia | FMNH206655 | 4.8 | 7.9 |
| *Hydrophis schistosus* | Malaysia | FMNH206657 | 5.5 | 7.4 |
| *Hydrophis schistosus* | Malaysia | FMNH206725 | 5.4 | 7.5 |
| *Hydrophis spiralis* | Phuket Thailand | ZMUC66844 | 5 | 8.5 |
| *Hydrophis spiralis* | Phuket Thailand | ZMUC66845 | 6 | 10.6 |
| *Hydrophis spiralis* | Phuket Thailand | ZMUC6646 | 5.4 | 8.9 |
| *Hydrophis spiralis* | Phuket Thailand | ZMUC66847 | 4.9 | 7.8 |
| *Hydrophis spiralis* | Malaysia | FMNH201042 | 4.2 | 7.3 |
| *Hydrophis spiralis* | Thailand | FMNH242122 | 3.2 | 5.3 |
| *Hydrophis spiralis* | Thailand | FMNH242123 | 4.4 | 7.8 |
| *Hydrophis stokesii* | Philippines | FMNH202826 | 4.5 | 5.4 |
| *Hydrophis stokesii* | Australia | SAMR65219 | 5.7 | 7.3 |
| *Hydrophis stokesii* | Australia | WAMR61734 | 7.7 | 10.5 |
| *Hydrophis stokesii* | Western Australia | WAMR174251 | 8.2 | 10.6 |
| *Hydrophis stricticollis* | Bay of Bengal | BMNH 68.4.3.42 | 3.3 | 6.2 |
| *Hydrophis stricticollis* | Bay of Bengal | NO LABEL, SAME JAR AS BMNH 68.4.3.41 | 2.9 | 5 |
| *Hydrophis stricticollis* | Bay of Bengal | BMNH 1908.6.23.82 | 2.9 | 6.5 |
| *Hydrophis stricticollis* | Bay of Bengal | BMNH 68.4.3.47 | 3.9 | 7.3 |
| *Hydrophis torquatus* | Malaysia | FMNH201169 | 5.3 | 8 |
| *Hydrophis viperinus* | Malaysia | FMNH201372 | 5.6 | 7.9 |
| *Hydrophis viperinus* | Malaysia | FMNH201373 | 5.7 | 7 |
| *Hydrophis viperinus* | Malaysia | FMNH201469 | 4.7 | 7.5 |
| *Hydrophis viperinus* | Malaysia | FMNH201476 | 5.9 | 8 |
| *Hydrophis viperinus* | Malaysia | FMNH201578 | 5.4 | 7 |
| *Hydrophis viperinus* | Malaysia | FMNH201588 | 5 | 5.9 |
| *Hydrophis viperinus* | Malaysia | FMNH201594 | 4.9 | 8 |
| *Hydrophis viperinus* | Malaysia | FMNH201598 | 6.4 | 7.4 |
| *Hydrophis zweifeli* | Australia | KLS_MW04690 | 3.5 | 4.5 |
| *Microcephalophis gracilis* | India Ocean | ZMUC R66400 | 3 | 8.1 |
| *Microcephalophis gracilis* | Thailand | FMNH178673 | 1.3 | 3.6 |
| *Microcephalophis gracilis* | Malaysia | FMNH201933 | 1.8 | 5.6 |
| *Microcephalophis gracilis* | Malaysia | FMNH201934 | 2 | 7.1 |
| *Microcephalophis gracilis* | India | FMNH210128 | 2.4 | 7.7 |
| *Microcephalophis gracilis* | Sri Lanka | KLS009 | 2 | 6.2 |
| *Parahydrophis mertoni* | Australia | MAGNTR00458 | 2.6 | 4.5 |

**Figure S1** BEAST maximum credibility tree for the concatenated mitochondrial and nuclear loci. Node labels show Bayesian posterior probabilities. Node bars show 95% highest posterior distributions for major divergences. Timescale is in millions of years before present.

**
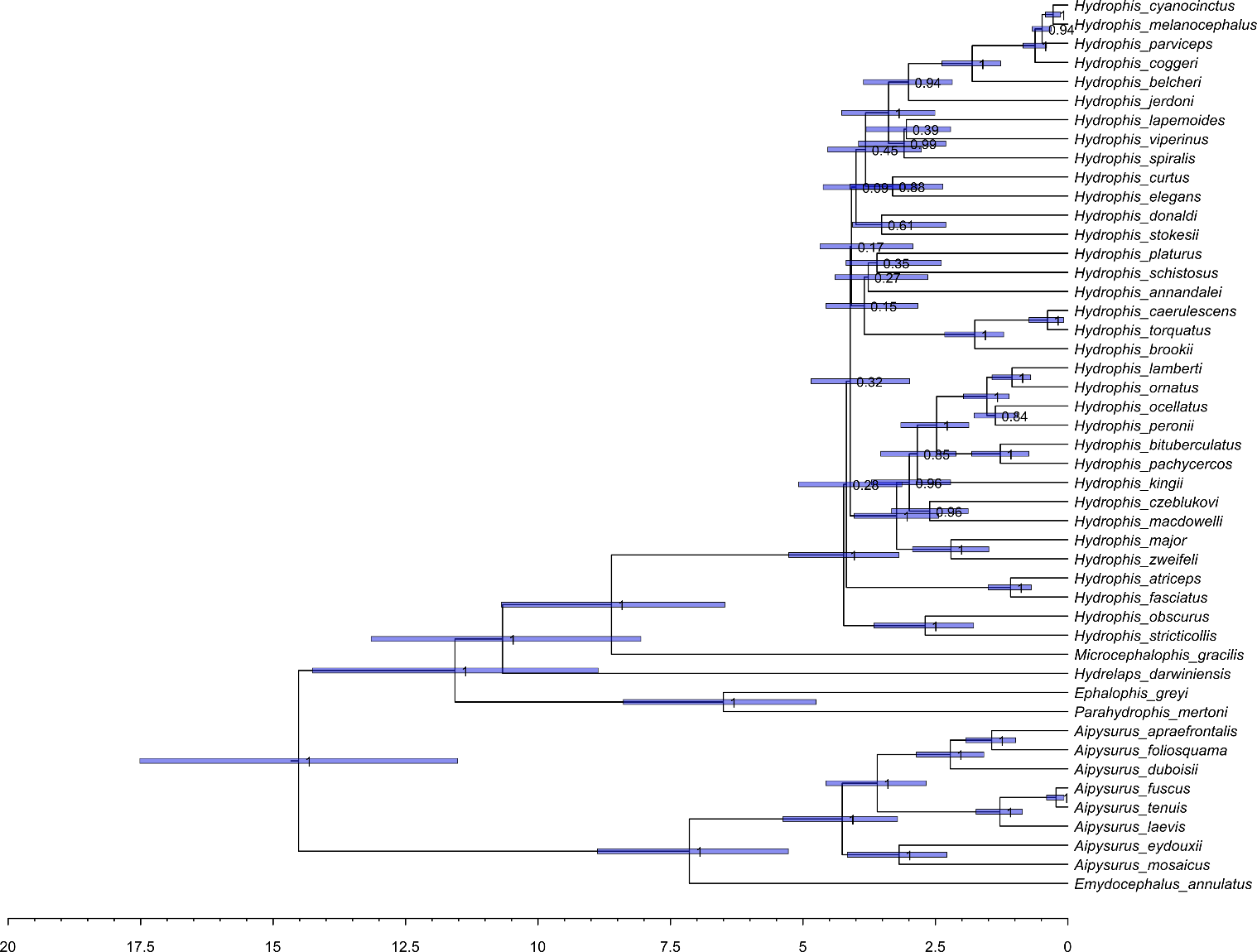
**

**Figure S2** Variation in A) relative girth and B) log-transformed maximum total length among the burrowing eel specialists, goby-eating species and other species that have less that 70% burrowing prey in their diet.


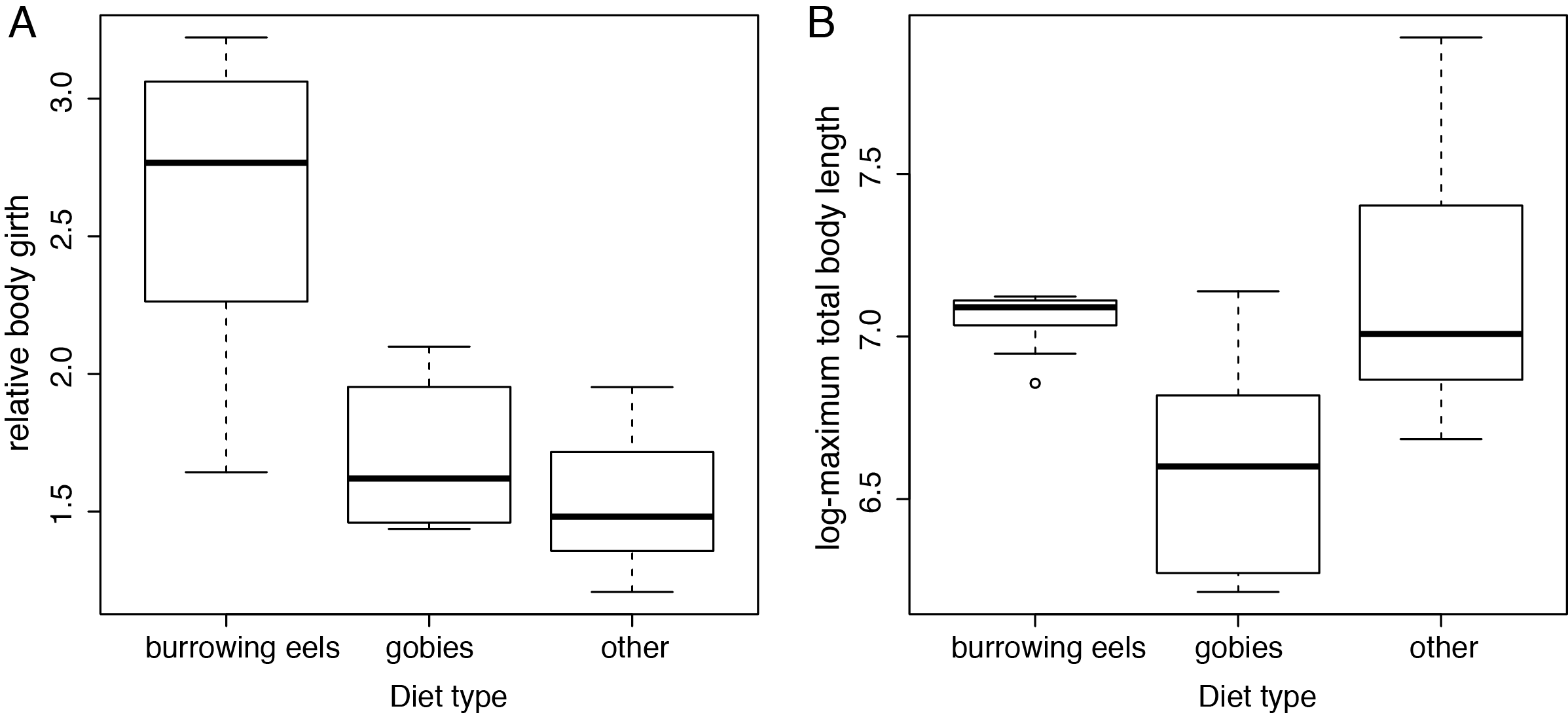


**Figure S3** Distribution of rates of morphological evolution (σ^2^) for A) relative girth and B) log-transformed maximum total length, compared among burrowing eel specialists, goby-eating species and other species, as estimated from a distribution of 500 trees. Mean estimates of σ^2^ for each group are shown as vertical line.


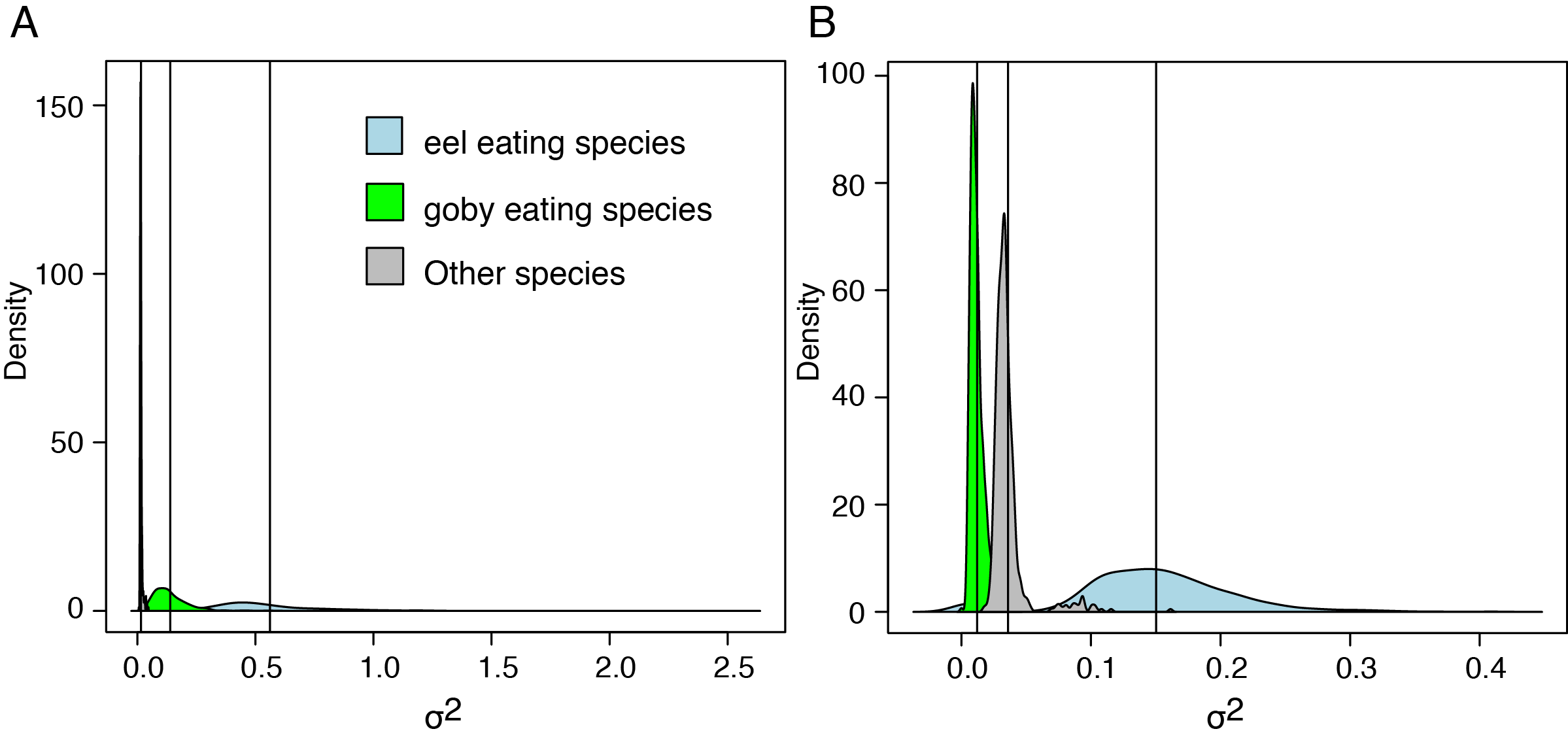

Supplement: Supplementary Materials [file rsos172141supp1.docx]
